# Chit-Chat

**Published:** 1873-10

**Authors:** 


### Editorial Chit Chat.

THE NEW BRIDGE.—The splendid iron bridge, across the river in this city, is completed, and is visited and admired by hundreds of people daily. The contractor, Mr. Shipman, has done us a good work, and has faithfully performed all, and more, than was asked of him. We have not the pleasure of his acquaintance, but we endorse and recommend him as a species of humanity not often encountered, viz: an honest contractor.

AN AMBULANCE.—Soon the ambulance of the Elmira Surgical Institute will be seen upon our streets, fitted up with a comfortable movable bed, ready to receive and convey to the Institute, any injured person in the city, who may desire to be attended by the physicians of this establishment.

Those who are poor and unable to pay, will receive treatment gratuitously. It is intended that all accidents occuring among the city's poor shall receive gratuitous treatment.

ANOTHER JOKE FROM H.—Since our last, H. has delivered himself of the following true story, which we regret we cannot relate in his inimitable style, yet we will try to preserve the text.

H. has a nephew, quite as fond of a joke as his honored uncle, and this is the story he tells of him: A farmer residing upon the farm of the nephew, called at the latter's banking house to transact some business, after the completion of which the nephew invited the old gentleman to dine with him. During the meal, the nephew passed a jar of olives to the farmer, asking him to take a "pickle." The farmer obeyed and, seeing the banker place a whole one in his mouth and disposing of it with evident gusto and relish, followed his example, much to his chagrin and disgust, for, having brought his molars in contact with the supposed pickle, but which can only be nauseating and disagreeable to a novice in gastronomic gymnastics, his face evinced evident disappointment, not to say anxiety. However, he slily slipped the olive into his hand and thence into his pocket, doubtless congratulating himself upon disposing of the supposed "spoiled pickle," without attracting the attention of his host. In this however he was estray also, for the nephew could not allow such an opportunity for quizzing the old farmer pass,—it was contrary to the laws of his nature, therefore did he modestly enquire of him what he intended doing with the olive? To this came the ready response of the farmer, to wit: "*will take it home to my wife, she never tasted of one, and I have!*"

DR. VON MOSCHZISKER.—We are happy to be able to testify to the scientific attainments and culture of this physician. Perhaps this recommendation will come with more force when it is considered that physicians are not apt to speak well of each other. Our intercourse with Dr. Von Moschzisker, has been of the most pleasant character, and we endorse him as a physician of unusual capabilities and acquirements.

BUSY.—If superintending the building of an hospital, attending a clinic of from forty to fifty patients daily, and editing a magazine, might be considered as likely to keep one person busy, perhaps it would be as well to give us credit accordingly, and forgive the evident hastiness with which this number of the BISTOURY has been prepared.

BALLOONING.—Prof. King, the aeronaut, recently sailed in view of this city, in his large balloon "Buffalo," *enroute* from the City of Buffalo. Mr. King seems to be the only aeronaut in this country who makes engagements and then fulfills them. We commend him to the *Graphic* folks, who, if they desire the success of their balloon enterprise, can be assured of a man who will certainly *try* to execute all he promises to.

We believe however, Prof. King has no faith in the project of crossing the Atlantic in a balloon. Of one thing we are assured, he would not promise such an expedition unless he meant to carry it out. In this particular he does not resemble Wise, to any remarkable extent.

FITNESS OF THINGS.—The bill boards in this city are ornamented with the bills of a traveling doctor, in which is exhibited the picture of a skull and inscription over it "death loves catarrh." We should call that a stunning card, and beg leave to express the opinion that if death is so confoundedly in love with catarrh that he can keep it, for aught we care.

Another Park.—Mayor Caldwell, Lormore Bros. & Mr. James Sly, are laying out a park and pleasant drives about the pond, upon their property in the 5th Ward. The bank of the pond is to be planted with willows, a road-way built about it and a rustic bridge thrown accross one end. Our citizens will then have another pleasant resort.

Swindling.—Some parties in Syracuse are sending out circulars purporting to come from Dr. E. R. Marlette, an oculist, who died several years ago in an insane asylum. As the doctor is defunct, he can't very well be engaged in the "free formula" business, proposing to cure catarrhal patients with his prescription, upon application, without money and without price.

Those who are silly enough to expect to get *something* for *nothing* will not only be disappointed, but swindled when they forward their $10 to these shrewd speculators who volunteer to send the formula already prepared, for the sum named, when it is found their druggist is unable to fill the prescription.

Don't, Please.—Dr. John B. Ogden, that contemptible fraud, who has his abiding place somewhere in the city of New York, and who advertises to send a formula for certain ailments, free of charge, and whose swindling operations we have frequently exposed, in common with other rascals of his ilk, writes us that unless we "let up" on him, he will "go for us," and make us "pay damages for injuring his business," etc.

Now, please don't John, you know you are a miserable, contemptible, swindling vagabond, and what under the sun is the use of becoming offended at us, just for hinting at the facts, in the very delicate manner and style that we invariably adopt for such humbugs as you. When yon "go for us" dear John, please use a breech loader, No. 10 shot, and at long range. For mercy sake, don't fire any of your free formulæ at us—we can stand anything but that. Sorry your letter did not reach us in season for the department devoted to such literature. Don't worry John, it isn't likely that we can make you out half as big a scamp as you really are, and then, you should have swindled enough green-horns with your free formula enterprise by this time, to retire to a brown stone front. Da, da, John.

Masonic Temple.—The masons have accepted plans and specifications for their temple, to be erected on corner of Lake and Market Sts. It will be the handsomest building in the city.

Disgraceful.—If you have occassion to seek the advice of a specialist in the practice of medicine, do not do violence to your own ideas of propriety, by calling upon any man who will so disgrace the profession of medicine as to defile the bill boards of your native town, with show-bills and wood-cuts that would do discredit to a fourth rate circus.—These are not the men that are apt to be eminent in their calling.

Malt Extract.—Some one has been kind enough to send to our address a case of "Dr. Lounsbury's Malt Extract." As we are not overstocked with confidence in anything resembling a "proprietory medicine," we approached the package with some misgivings as to the propriety of even tasting it.

We finally did so however, and have found sufficient confidence in its use to recommend it as really a very excellent and pure malt extract, suitable for a tonic or pleasant beverage.

Sad Death.—Dr. P. R. Wagenseller, an eminent physician, of Selins Grove, Pa., was recently stabbed and killed, by a drunken vagabond of that town, recently. Dr. Wagenseller was a highly cultured and intellectual gentleman whom it was an honor and pleasure to know. Many are the pleasant hours of professional chat we have had with him, in which he always exhibited himself a well-read and remarkably gifted man in his profession of medicine and surgery. We deplore his loss, and that so valuable a life should have been sacrificed at the hands of a miserable and worthless scoundrel.

Baby Farming.—A shocking and outrageous instance of "baby farming" was recently brought to the notice of the court of Special Sessions, in New York. It seems that a Mrs. Coates, residing at No. 205 E. 63d St. had been in the habit of placing an infant in a basket and then allowing it to remain unsheltered from the sun, in the back yard of her premises, during the entire day. Neighbors were apprised of this cruel treatment of the babe for several days before information was given to the police. The babe died and the woman was arrested, saying that she had six other infants in her charge which she gave "fresh air" daily, in the same manner, as it "was good for them." The child that died proved to have belonged to Barnum's "Circassian girl." The court sentenced the woman Coates, to three months imprisonment in the penitentiary. She was led away, hypocritically protesting her innocence, in a flood of tears. Poor woman! An oven, at a temperature of 210°, or thereabout, would have become her better.

PROPRIETORY MEDICINES.—The regular medical press would scorn to publish advertisements of "Drake's Plantation Bitters," Helmbold's Buchu, etc., because they are so-called "patent medicines" and put up to *sell* as cure-alls. These same journals do not flinch from giving widespread publicity to equally absurd and worthless compounds, when such advertisements are offered by the "regular profession." Look at the advertising pages of any respectable medical journal, and you will find them crammed with advertisements of medical compounds of various and multitudious forms, recommended for as many ailments as the quack medicines first referred to. We have the "Elixir-Iodo-Bromide of Calcium Compound," that is a sure cure for "scrofula, swellings, throat affections, salt rheum, scald head," etc., etc., and will find the advertisement of this wonderful cure-all accompanied by the certificates of numerous respectable practitioners, who have cured all manner of disease by its use.

Then you will find the elixirs of iron, lime, soda, potassa, and calisaya, variously combined, and recommended for every disease under the sun. These elixirs have become so fashionable a prescription among the fraternity, that you can scarcely enter a sick room without having a bottle labeled "Ferro-phos—elixir-calisaya and strychnia," or some equally ridiculous combination of sweetened cherry, minus any of the other ingredients mentioned, in the label, staring you in the face. When physicians have to rely upon pharmacists to prescribe for their patients, as well as furnish the remedies, we see but little if any use for the doctor. Our opinion, based upon a pretty thorough examination of elixirs and other proprietory preparations, is that they are useless, worthless, and unmitigated frauds. The less the profession have to do with them, the less disappointment they will encounter in their effects.

